# GLUT3 regulates alternative macrophage signaling through a glucose transport–independent role

**DOI:** 10.1172/JCI174540

**Published:** 2023-11-01

**Authors:** Peng Zhang, Jason Miska, Amy B. Heimberger

**Affiliations:** Department of Neurological Surgery, Malnati Brain Tumor Institute, Northwestern University Feinberg School of Medicine, Chicago, Illinois, USA.

## Abstract

Macrophages are key mediators of innate immunity whose functional state can be regulated by glucose transporters. Although abundantly expressed in macrophages, the specific function of GLUT3, an isoform of facilitative glucose transporters, has not been clearly established. In this issue of the *JCI*, Dong-Min Yu and colleagues identify an alternative role for GLUT3 in promoting M2 macrophage polarization. The authors demonstrated that GLUT3 was upregulated upon M2 stimulation and was required for efficient alternative macrophage polarization and function. They further showed that GLUT3-induced M2 polarization was independent of glucose transport and functioned through Ras-mediated regulation of IL-4R endocytosis and IL-4/STAT6 activation. These findings may guide the development of macrophage-targeted treatments.

## Role of glucose transporters in macrophage function

Macrophages are key mediators of innate immunity, widely distributed throughout the body, and play an important role in regulating inflammation, tissue homeostasis, and pathogen clearance. As a heterogeneous population of myeloid cells, macrophages are well known for their highly diverse and plastic features (1). These cells rapidly respond to environmental cues or stimuli to reshape their morphology, phenotype, and functionality. An M1/M2 polarization paradigm has been broadly used to define two distinct subtypes of macrophages, i.e., M1 or proinflammatory/classically activated macrophages and M2 or antiinflammatory/alternatively activated macrophages. However, this oversimplified dichotomization is an in vitro phenomenon that does not portray the full spectrum of these cells under complex scenarios (2). Despite these limitations, this paradigm has provided crucial and biologically relevant insight into the role of macrophages in the context of inflammation, tissue repair, autoimmune diseases, and tumors. Importantly, this model has led to potential actionable strategies that reprogram or repolarize macrophages as promising therapeutic approaches for various diseases (3). As such, there is a need for an in-depth understanding of macrophage polarization mechanisms.

Accumulating immunometabolism studies have characterized the correlation between the metabolic features of macrophages and their functions (4), thereby highlighting the key role of glycolysis in regulating functional states. Glycolysis converts glucose into pyruvate for production of ATP and other intermediate products for the biosynthesis of amino acids and nucleotides (5). Several families of glucose transporters have been identified, including facilitative sugar transporters (GLUTs), Na^+^/glucose cotransporters (SGLTs), and sugars will eventually be exported transporters (SWEETs) (6). GLUTs are also known to regulate the immune function of macrophages. For example, overexpression of GLUT1 largely increased glucose uptake and metabolism, thus enhancing the secretion of inflammatory mediators and inflammatory function of macrophages (7). There are 13 GLUT isoforms, which have different features regarding tissue distribution, subcellular localization, and function (8). Although some GLUT isoforms, such as GLUT1, have been intensively studied, further studies deciphering the specific function of other GLUT isoforms, particularly in immune cells, await future study.

## GLUT3 is essential to M2 macrophage phenotypes

Sharing 65%–66% sequence identity (9), GLUT1 and GLUT3 are both abundantly expressed in macrophages (10), but there is limited knowledge about their specific roles in modulating immune functions of these cells. To address this knowledge gap, Dong-Min Yu and colleagues aimed to investigate (i) how the expression of these GLUTs responds to polarization stimuli, and (ii) what effects these GLUT isoforms have on polarization and function of macrophages (11). By using a variety of models, including murine bone marrow–derived macrophages (BMDMs), RAW 264.7 cells, human THP-1 cells, and peripheral blood–derived monocytes, the authors demonstrated that GLUT1 and GLUT3 were upregulated by M1 and M2 stimuli, respectively. They also generated myeloid cell–specific GLUT1-KO and GLUT3-KO mice to demonstrate that GLUT3 deficiency impaired M2 polarization of macrophages. Unlike GLUT1, GLUT3 did not appear to be the major contributor to glucose uptake and metabolism in macrophages, and the deletion of macrophage GLUT3 did not lead to compensatory upregulation of other glucose transporters. To gain further insights into the functional role of GLUT3 in M2 macrophages in vivo, Yu et al. used human tissues and mouse models to illustrate that GLUT3 promoted allergic inflammation and wound healing functions (11).

**Figure 1:**
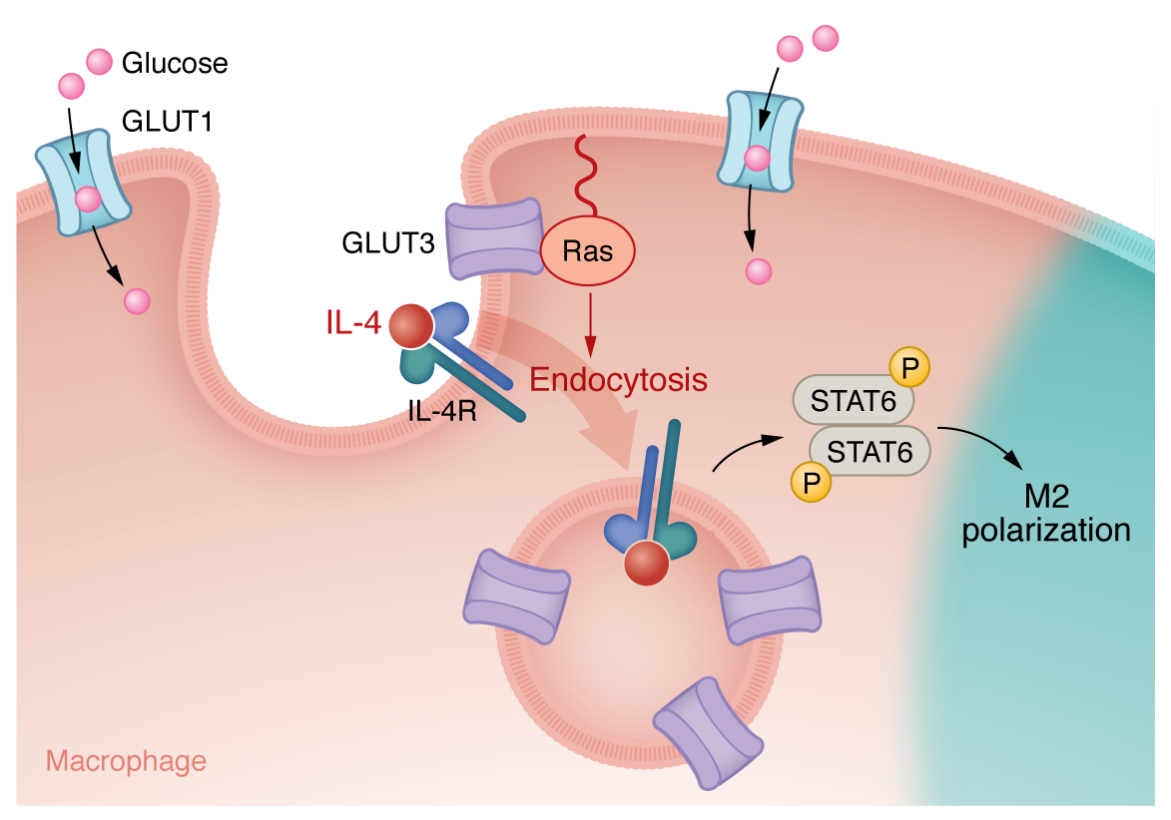
GLUT3 induces M2 polarization of macrophages. GLUT3-induced M2 polarization is glucose transport independent and functions through Ras-mediated regulation of IL-4R endocytosis and IL-4/STAT6 activation.

Mechanistically, the investigators showed that GLUT3 mediated IL-4/STAT6 signal transduction (Figure 1) — a key signaling pathway for M2 polarization (12). They showed reduced phosphorylated STAT6 (p-STAT6) in response to M2 stimulation in GLUT3-KO cells, but not impaired p-STAT1 during M1 stimulation, highlighting the role of GLUT3 in M2 polarization through STAT6 signaling. Using both genetic and pharmacological manipulation with G3iA, a GLUT3 glucose transport inhibitor, they clarified that GLUT3-mediated STAT6 activation and M2 polarization did not rely on its glucose transport activity.

Since GLUT isoforms have different subcellular localizations, Yu et al. confirmed that GLUT3 is predominantly localized intracellularly using immunofluorescence and cell fractionation (11, 13). Based on the endosomal colocalization of GLUT3 and p-STAT6 after IL-4 stimulation and the fact that the STAT6 activation was impaired with an inhibitor of dynamin and endocytosis, the authors posited that endocytosis is essential for p-STAT6 induction after IL-4 stimulation. This hypothesis was confirmed based on the finding that GLUT3 deficiency impaired endocytosis but did not affect the expression of IL-4 receptor α (IL-4Rα) or common gamma (γ_c_) chain, the heterodimers forming type I IL-4Rs. To understand how endosomal GLUT3 regulated IL-4R endocytosis, Yu et al. performed binding studies that found a direct interaction between the intracytoplasmic loop (ICH) domain of GLUT3 with GTP-bound Ras, which promotes IL-4R subunit endocytosis, STAT6 signaling, and M2 polarization (11).

## Conclusions and future directions

Yu et al. demonstrate the essential role of GLUT3 in polarization and function of M2 macrophages, and shed light on the underlying mechanisms. The authors conclude that endosomal GLUT3 promotes IL-4/STAT6 signaling by directly interacting with Ras and regulating IL-4R endocytosis. One highlight of this work is the identification of a unique and critical role of GLUT3 in macrophage polarization and function, which does not rely on its glucose transport function and glycolysis in macrophages. GLUT3 is a glucose transporter that is abundantly expressed in macrophages, but its specific function, particularly in macrophages or in general immune cell populations, remains to be fully understood.

The results from Yu et al. (11) may have high scientific value and clinical importance, considering the essential function and high abundance of macrophages in various diseases. The subcellular localization of GLUT3 as well as its function in endosomal signaling and cytokine receptor signaling pathways, as revealed by this study, may open a door for future studies to explore the role of GLUT3 in regulating other receptor tyrosine kinases that also require endocytosis and endosomal enrichment for signal transduction (11). It would be intriguing to explore which cytokines and signaling pathways require GLUT3 function for optimal signal transduction. More specifically, is GLUT3 solely involved in antiinflammatory or immunosuppressive cytokine-mediated signaling? Areas of future investigation also include the function of GLUT3 in other cells such as neurons (14), tumor cells, and even other myeloid populations like microglia and tumor-associated macrophages. For example, Tsai et al. reported that GLUT3 is highly expressed in triple-negative breast cancer cells (15). This expression is correlated with a proinflammatory gene signature and activation of M1 tumor-associated macrophages through GLUT3-regulated tumor secretion of CXCL8 (15). As the crucial role of the myeloid compartment in the tumor microenvironment has emerged and gained increasing importance, how GLUT3 expression and GLUT3-targeted therapy affect tumor progression and immunotherapy outcome may be clinically relevant. This work by Yu et al. (11) may provide valuable clues for guiding the development of new macrophage-targeted therapy with potential clinical benefits. Other than GLUT3, this study may also inspire future studies to explore the unknown functions of other GLUT isoforms in addition to/rather than glucose transport.

## Version 1 11/01/2023

Electronic publication
